# Association of Sodium-Glucose Cotransporter 2 Inhibitor vs Dipeptidyl Peptidase-4 Inhibitor Use With Risk of Incident Obstructive Airway Disease and Exacerbation Events Among Patients With Type 2 Diabetes in Hong Kong

**DOI:** 10.1001/jamanetworkopen.2022.51177

**Published:** 2023-01-17

**Authors:** Philip C. M. Au, Kathryn C. B. Tan, David C. L. Lam, Bernard M. Y. Cheung, Ian C. K. Wong, Wang Chun Kwok, Chor-Wing Sing, Ching-Lung Cheung

**Affiliations:** 1 Department of Pharmacology and Pharmacy, Li Ka Shing Faculty of Medicine, University of Hong Kong, Pokfulam, Hong Kong Special Administrative Region, China; 2 Department of Medicine, Li Ka Shing Faculty of Medicine, University of Hong Kong, Pokfulam, Hong Kong Special Administrative Region, China; 3 Laboratory of Data Discovery for Health, Hong Kong Science Park, Pak Shek Kok, Hong Kong Special Administrative Region, China; 4 Research Department of Practice and Policy, School of Pharmacy, University College London, London, United Kingdom

## Abstract

**Importance:**

Patients with diabetes are at higher risk for obstructive airway disease (OAD). In recent meta-analyses of post hoc analyses of cardiorenal trials, sodium-glucose cotransporter 2 inhibitors (SGLT2Is) were suggested to reduce the risk of OAD adverse events. However, a clinical investigation of this association is warranted.

**Objective:**

This study aimed to investigate the association of SGLT2I use vs dipeptidyl peptidase-4 inhibitor (DPP4I) use with OAD incidence and exacerbation events in patients with type 2 diabetes.

**Design, Setting, and Participants:**

This retrospective population-based cohort study used electronic health data from a territory-wide electronic medical database in Hong Kong. Data were collected for patients with type 2 diabetes who were prescribed SGLT2Is or DPP4Is between January 1, 2015, and December 31, 2018. Patients were followed for a median of 2.2 years between January 1, 2015, and December 31, 2020. A prevalent new-user design was adopted to match patients based on previous exposure to the study drugs. Propensity score matching was used to balance baseline characteristics.

**Exposures:**

Patients with type 2 diabetes using SGLT2Is (exposure of interest) or DPP4Is (active comparator).

**Main Outcomes and Measures:**

The main outcomes were the first incidence of OAD and the count of OAD exacerbations. The risk of incident OAD was estimated using a Cox proportional hazards regression model. The rate of exacerbations was estimated using zero-inflated Poisson regression. Statistical analysis was performed on November 13, 2022.

**Results:**

This study included 30 385 patients. The propensity score–matched non-OAD cohort (incidence analysis) consisted of 5696 SGLT2I users and 22 784 DPP4I users, while the matched OAD cohort (exacerbations analysis) comprised 381 SGLT2I users and 1524 DPP4I users. At baseline, 56% of patients in the non-OAD cohort were men and the mean (SD) age was 61.2 (9.9) years; 51% of patients in the OAD cohort were men and the mean age was 62.2 (10.8) years. Compared with DPP4I use, SGLT2I use was associated with a lower risk of incident OAD (hazard ratio, 0.65 [95% CI, 0.54-0.79]; *P* < .001) and a lower rate of exacerbations (rate ratio, 0.54 [95% CI, 0.36-0.83]; *P* = .01). The associations were consistent in sex subgroup analysis.

**Conclusions and Relevance:**

The findings of this retrospective cohort study of patients with type 2 diabetes in Hong Kong suggest that SGLT2I use was associated with a reduced risk of incident OAD and a lower rate of exacerbations in a clinical setting compared with DPP4I use. These findings further suggest that SGLT2Is may provide additional protective effects against OAD for patients with type 2 diabetes and that further investigation is warranted.

## Key Points

**Question:**

Are sodium-glucose cotransporter 2 inhibitors (SGLT2Is) associated with risk of incident obstructive airway disease (OAD) and exacerbation events in clinical settings among patients with type 2 diabetes compared with dipeptidyl peptidase-4 inhibitors (DPP4Is)?

**Findings:**

In this cohort study using electronic health data for 30 385 patients with type 2 diabetes in Hong Kong, we found that SGLT2I use was associated with a 35% reduced risk of incident OAD and a 46% reduced rate of exacerbations compared with DPP4I use.

**Meaning:**

These findings suggest that SGLT2I use may provide additional protective effects against OAD for patients with type 2 diabetes and encourage further investigation.

## Teaser

This cohort study examines the association of sodium-glucose cotransporter 2 vs dipeptidyl peptidase-4 inhibitor use with the incidence of obstructive airway disease and exacerbation events in patients with type 2 diabetes.

## Introduction

Chronic respiratory diseases are the third leading cause of death, after cardiovascular diseases and cancers.^1^ Among the chronic respiratory diseases, obstructive airway disease (OAD), including chronic obstructive pulmonary disease (COPD) and asthma, is the most common cause of death.^1^ Diabetes is a common comorbidity of OAD.^2,3^ Previous studies reported that OAD was associated with a higher risk of diabetes,^4,5^ and diabetes was associated with a higher risk of OAD^6,7^ and exacerbation events.^8^ Given the interrelated nature of OAD and diabetes, it is of clinical importance to investigate the effects of antidiabetic medications on OAD.

Sodium-glucose cotransporter 2 inhibitors (SGLT2Is) are a novel class of antidiabetic medications that confer protective effects on the cardiovascular system and kidneys and possibly the respiratory system.^9,10,11,12^ Animal studies have shown SGLT2Is to inhibit NLR pyrin domain containing protein 3 (NLRP3) inflammasome activation in multiple tissues, including the heart,^13^ liver,^14^ kidney,^15^ and lung.^16^ Inhibition of the NLRP3 inflammasome has been implicated in both improved asthmatic airway inflammation^17^ and COPD exacerbations.^18^ Therefore, SGLT2Is may potentially affect OAD. However, to our knowledge, there are no available studies that directly evaluate this association. A recent meta-analysis of cardiorenal trials reported that compared with placebo, SGLT2I use was associated with a reduced risk of OAD.^19^ A network meta-analysis further suggested that SGLT2I use was associated with a reduced incidence of asthma compared with dipeptidyl peptidase-4 inhibitor (DPP4I) use.^20^ However, the OAD events in these trials were likely underreported, as they were extracted from reports of serious adverse events rather than being the primary end points. The study populations were also highly selected with a low risk of OAD. Moreover, only an indirect comparison of SGLT2Is and DPP4Is was provided in the network meta-analysis. Therefore, the findings from these meta-analyses were hypothesis generating in nature, and it remains unclear whether similar associations would be observed in clinical settings. This retrospective cohort study aimed to investigate the clinical association of SGLT2I use vs DPP4I use with incident OAD and exacerbation events among patients with type 2 diabetes in Hong Kong.

## Methods

Ethical approval for this cohort study was granted by the University of Hong Kong/Hospital Authority (HA) Hong Kong West Cluster Institutional Review Board, which waived the need for informed consent from study participants owing to the use of anonymized electronic health record data. The study followed the Strengthening the Reporting of Observational Studies in Epidemiology (STROBE) reporting guideline.

### Data Source

The Clinical Data Analysis and Reporting System (CDARS) is a territory-wide representative electronic medical database from Hong Kong HA. Details on the CDARS are provided in the eMethods in Supplement 1.

### Study Cohort

The study cohort consisted of patients with diabetes who were prescribed SGLT2Is or DPP4Is between January 1, 2015 (the year SGLT2Is were first available for prescription under the HA), and December 31, 2018. Patients who started any SGLT2I were classified as the exposed group, whereas those who started any DPP4I but had not been prescribed any SGLT2Is previously were classified as the control group. The exclusion criteria are provided in the eMethods in Supplement 1. For the disease incidence analysis, patients were excluded if they had a history of OAD diagnosis or any filled prescriptions for an inhaled corticosteroid (ICS), bronchodilator (including a long- or short-acting β-agonist [LABA/SABA], long- or short-acting muscarinic antagonist [LAMA/SAMA], or theophylline), leukotriene receptor antagonist (LTRA), or phosphodiesterase-4 inhibitor (PDE4I) 1 year before the index date. For the exacerbation analysis, only patients excluded from the incidence analysis were included. The cohort was followed until the occurrence of study outcomes (for the incidence analysis only), study end (December 31, 2020), death, index drug discontinuation, or switch to or addition of an SGLT2I (for the DPP4I group only), whichever came first. Discontinuation was defined as more than 90 days without a new prescription after the end of the last prescription.

### Outcomes

There were 2 outcomes of interest: the first incidence of OAD and the counts of OAD exacerbations. Incident OAD was defined as the first clinical diagnosis of OAD (*International Classification of Diseases, Ninth Revision, Clinical Modification* [*ICD-9-CM*] codes 491.x-496.x) from outpatient, emergency department, or inpatient records or as the first filled prescription of an ICS, bronchodilator (including a LABA/SABA, LAMA/SAMA, or theophylline), LTRA, or PDE4I after the index date. An OAD exacerbation event was defined as an emergency department or inpatient visit with a diagnosis of OAD or as a prescription of short-course (<7 days) oral corticosteroids from an emergency department or inpatient visit.^21,22^ Exacerbation events within 7 days were considered a single event.^23^

### Prevalent New-User Design

Since many patients who started SGLT2Is were ongoing or previous users of DPP4Is (ie, prevalent users), this study adopted the prevalent new-user design to account for prior exposure to an active comparator.^24^ A detailed explanation of how the design was applied to the study cohort is described elsewhere^12^ and in the eMethods in Supplement 1.

### Propensity Score Matching

Propensity score (PS) matching^25^ was adopted to balance baseline characteristics of the exposed and control groups. We selected a comprehensive list of covariates for PS calculation, including age, sex, estimated glomerular filtration rate, hemoglobin A_1c_ measurements, calendar season of index date, clinical visits, previous and concurrent use of other glucose-lowering agents, and histories of major comorbidities and related drug uses (a full list appears in eTable 2 in Supplement 1). Covariates were assessed 1 year before the index date. For the exacerbation analysis, histories of bronchiectasis and extrinsic allergic alveolitis, bronchodilator and ICS use (including corticosteroid/bronchodilator/LABA combinations), and levels of maintenance therapy for OAD 1 year before the index date were also included to account for potential heterogeneity within the OAD population. Methods for PS calculation and matching are provided in the eMethods in Supplement 1. The balance of covariates after PS matching was assessed with standardized mean differences (SMDs). Covariates with an SMD greater than 0.1 were considered unbalanced and were adjusted in subsequent regression analyses.

### Statistical Analysis

Patient characteristics are presented as means (SDs) for continuous variables and frequencies (percentages) for categorical variables. For the incidence OAD analysis, hazard ratios (HRs) with 95% CIs were estimated using a Cox proportional hazards regression model. Absolute risk differences (ARDs) at 1 year of follow-up were estimated using the method proposed by Austin,^26^ in which survival probabilities were averaged across patients and the cumulative incidence was estimated as follows: 1 – mean(survival probability). The number needed to treat (NNT) was estimated as 1/ARD. For the OAD exacerbation analysis, rate ratios (RRs) of exacerbation counts were estimated using a zero-inflated Poisson (ZIP) regression model.^23^ Excess zero counts of an event are a potential bias arising from patients with zero likelihood (ie, not at risk) of having an event. The ZIP model accounts for this bias by fitting a logit (zero) model, which predicts the odds of having excess zero counts of an event, with a Poisson (count) model, which estimates the rate of exacerbation events. Statistical significance was defined as 2-sided *P* ≤ .05. All statistical analyses were performed using R, version 4.1.0 (R Foundation for Statistical Computing). Statistical analysis was performed on November 13, 2022.

### Sensitivity Analysis

#### Incidence Analysis

Two sensitivity analyses with more stringent definitions for incident OAD were conducted. The first one counted a prescription of a short-acting bronchodilator as an outcome event only when it was prescribed for 7 days or longer. The second one excluded any outcome events when patients were diagnosed with acute bronchitis (*ICD-9-CM* code 466.x or 490.x) or heart failure (*ICD-9-CM* codes 398.91, 402.01, 402.11, 402.91, 404.01, 404.11, 404.91, or 428) at any time during follow-up.

#### Exacerbation Event Analysis

To capture patients who were unable to tolerate oral corticosteroids, a sensitivity analysis was conducted by including a prescription of injected corticosteroids from an emergency department or inpatient visit as an OAD exacerbation event. To account for potential misdiagnosis of heart failure symptoms as OAD exacerbations, another sensitivity analysis that excluded any exacerbation events when patients were diagnosed with heart failure at any time during follow-up was also conducted.

### Subgroup Analysis

Subgroup analysis was performed to evaluate the association of SGLT2I use vs DPP4I use with incident OAD in subgroups of men and women, separately. Since men and women had different baseline function, PS was recalculated and rematched within the subgroups. Interactions were tested by adding the corresponding interaction term to the Cox regression of the main (full cohort) analysis.

## Results

This study included 30 385 patients. There were 5696 and 381 SGLT2I users and 22 784 and 1524 DPP4I users in the final non-OAD and OAD PS-matched cohorts, respectively (eFigure 1 in Supplement 1). At baseline, 56.9% of patients in the non-OAD cohort were men and 44.1% were women (mean age [SD] of 61.2 [9.9] years; Table 1); 51.0% of patients in the OAD cohort were men and 49.0% were women (mean [SD] age of 62.2 [10.8] years; Table 2). In the non-OAD cohort, 2279 SGLT2I users (40.0%) were new users and 3417 (60.0%) were prevalent users. In the OAD cohort, 175 SGLT2I users (46.0%) were new users and 206 (54.0%) were prevalent users. Baseline characteristics of the 2 matched cohorts are presented in Tables 1 and 2. All covariates had an SMD of less than 0.1 after PS matching except for the previous use of lipid-regulating agents (SMD = 0.10) in the OAD cohort (Tables 1 and 2), which was adjusted in subsequent regression analyses.

**Table 1.** Baseline Characteristics of the Cohort Without Obstructive Airway Disease After Propensity Score Matching

Covariate	No. of patients (%)		SMD
	DPP4I group (n = 22 784)	SGLT2I group (n = 5696)	
**Demographic**			
Sex			
Men	12 730 (55.9)	3208 (56.3)	0.01
Women	10 054 (44.1)	2488 (43.7)	
Age at index date, y, mean (SD)	61.2 (9.9)	61.0 (9.7)	0.02
Season of index date			
Winter (Dec-Feb)	5370 (23.6)	1342 (23.6)	0.01
Spring (Mar-May)	5700 (25.0)	1415 (24.8)	
Summer (Jun-Aug)	5677 (24.9)	1400 (24.6)	
Fall (Sept-Nov)	6037 (26.5)	1539 (27.0)	
**Medication history (1 y prior)**			
Cardiovascular			
Angiotensin-converting enzyme inhibitor/angiotensin II receptor blocker	15 665 (68.8)	3901 (68.5)	0.01
Antiarrhythmic agent	111 (0.5)	32 (0.6)	0.01
Anticoagulant	745 (3.3)	195 (3.4)	0.01
β-Blocker	8633 (37.9)	2179 (38.3)	0.01
Calcium channel blocker	11 240 (49.3)	2764 (48.5)	0.02
Cardiac glycoside	215 (0.9)	55 (1.0)	0.002
Loop diuretic	1057 (4.6)	274 (4.8)	0.01
Other diuretic	2181 (9.6)	514 (9.0)	0.02
Nitrate	2536 (11.1)	701 (12.3)	0.04
Peripheral vasodilator	96 (0.4)	26 (0.5)	0.01
Platelet inhibitor	7287 (32.0)	1870 (32.8)	0.02
Respiratory			
Bronchodilator	0	0	<0.001
Inhaled corticosteroid	0	0	<0.001
Psychotropic			
Antidepressant	1338 (5.9)	321 (5.6)	0.01
Antipsychotic	595 (2.6)	132 (2.3)	0.02
Immune related			
Antibiotics	3248 (14.3)	881 (15.5)	0.03
Immunosuppressant	171 (0.8)	47 (0.8)	0.01
Nonsteroidal or anti-inflammatory agent	3011 (13.2)	776 (13.6)	0.01
Kidney			
Phosphate-binding agent	0	0	<0.001
Other			
Lipid-regulating agent	18 331 (80.5)	4581 (80.4)	0.001
Proton pump inhibitor	3970 (17.4)	1086 (19.1)	0.04
Systemic corticosteroid	1043 (4.6)	275 (4.8)	0.01
Hormone replacement therapy	10 (0)	3 (0.1)	0.004
Glucose-lowering agent			
Metformin	19 758 (86.7)	4972 (87.3)	0.02
Sulfonylurea	16 326 (71.7)	4020 (70.6)	0.02
Thiazolidinedione	2495 (11.0)	512 (9.0)	0.07
Glucagon-like peptide-1 agonist	14 (0.1)	0	0.04
Acarbose	644 (2.8)	184 (3.2)	0.02
Meglitinide	0	0	<0.001
Insulin	2888 (12.7)	767 (13.5)	0.02
**Diagnosis history (1 y prior)**			
Cardiovascular			
Coronary heart disease	2184 (9.6)	613 (10.8)	0.04
Heart failure	0	0	<0.001
Myocardial infarction	507 (2.2)	148 (2.6)	0.02
Cerebrovascular	820 (3.6)	207 (3.6)	0.002
Hypertensive disease	4022 (17.7)	1047 (18.4)	0.02
Arrhythmia or conduction disorder	654 (2.9)	160 (2.8)	0.004
Arterial disease	305 (1.3)	80 (1.4)	0.01
Kidney			
Chronic kidney disease	1951 (8.6)	481 (8.4)	0.004
Respiratory			
Pneumonia	70 (0.3)	18 (0.3)	0.002
Acute bronchitis	14 (0.1)	4 (0.1)	0.003
Bronchiectasis	0	0	<0.001
Extrinsic allergic alveolitis	0	0	<0.001
Other lung disease	1213 (5.3)	303 (5.3)	<0.001
Metabolic and endocrine			
Obesity	851 (3.7)	225 (4.0)	0.01
Hyperlipidemia	2461 (10.8)	660 (11.6)	0.03
Thyroid disease	201 (0.9)	45 (0.8)	0.01
Osteoporosis	46 (0.2)	10 (0.2)	0.01
Osteoporotic fracture	85 (0.4)	18 (0.3)	0.01
All fractures	224 (1.0)	54 (0.9)	0.004
Paget disease	4 (0)	1 (0)	<0.001
Rheumatoid arthritis	0	0	<0.001
Cancer	470 (2.1)	119 (2.1)	0.002
Other			
Chronic pancreatitis	3 (0)	1 (0)	0.004
Dementia	33 (0.1)	6 (0.1)	0.01
Liver disease (chronic liver disease, cirrhosis, esophageal varix, or hepatic failure)	81 (0.4)	19 (0.3)	0.004
Diabetes related			
Diabetic eye complication	1869 (8.2)	455 (8.0)	0.01
Diabetic hyperosmolarity	13 (0.1)	2 (0)	0.01
Diabetic neuropathy	405 (1.8)	117 (2.1)	0.02
Peripheral artery disease	129 (0.6)	38 (0.7)	0.01
Diabetic ketoacidosis	14 (0.1)	5 (0.1)	0.01
Biochemical parameter, mean (SD)			
Hemoglobin A_1c_, %	8.52 (1.68)	8.59 (1.36)	0.05
eGFR, mL/min/1.73 m^2^	87.68 (20.98)	87.83 (18.81)	0.01
Clinical history, mean (SD)			
No. of glucose-lowering agents used, 5 y prior^a^	1.95 (0.64)	1.92 (0.66)	0.04
Days since first diabetes diagnosis	3324 (1748)	3264 (1784)	0.03
No. of emergency department admissions, 1 y prior	0.16 (0.47)	0.18 (0.52)	0.03
No. of planned admissions, 1 y prior	0.23 (0.83)	0.27 (0.96)	0.03
Comedication at baseline			
Metformin	19 085 (83.8)	4752 (83.4)	0.01
Sulfonylurea	14 499 (63.6)	3604 (63.3)	0.01
Thiazolidinedione	2024 (8.9)	416 (7.3)	0.06
Glucagon-like peptide-1 agonist	2 (0)	0	0.01
Acarbose	419 (1.8)	125 (2.2)	0.03
Meglitinide	0	0	<0.001
Insulin	7924 (34.8)	1973 (34.6)	0.003
Total No. of comedications, mean (SD)	1.93 (0.70)	1.91 (0.73)	0.03

Abbreviations: DPP4I, dipeptidyl peptidase-4 inhibitor; eGFR, estimated glomerular filtration rate; SGLT2I, sodium-glucose cotransporter 2 inhibitor; SMD, standardized mean difference.

^a^ Metformin, sulfonylureas, meglitinides, glucagon-like peptide receptor agonists, acarbose, and thiazolidinediones.

**Table 2.** Baseline Characteristics of the Cohort With Obstructive Airway Disease After Propensity Score Matching

Covariate	No. of patients (%)		SMD
	DPP4I group (n = 1524)	SGLT2I group (n = 381)	
**Demographic**			
Sex			
Men	778 (51.0)	198 (52.0)	0.02
Women	746 (49.0)	183 (48.0)	
Age at index date, y, mean (SD)	62.3 (10.8)	62.2 (10.7)	0.004
Season of index date			
Winter (Dec-Feb)	359 (23.6)	88 (23.1)	0.02
Spring (Mar-May)	399 (26.2)	102 (26.8)	
Summer (Jun-Aug)	394 (25.9)	98 (25.7)	
Fall (Sept-Nov)	372 (24.4)	93 (24.4)	
**Medication history (1 y prior)**			
Cardiovascular			
Angiotensin-converting enzyme inhibitor/angiotensin II receptor blocker	1030 (67.6)	266 (69.8)	0.05
Antiarrhythmic agent	27 (1.8)	8 (2.1)	0.02
Anticoagulant	89 (5.8)	25 (6.6)	0.03
β-Blocker	481 (31.6)	121 (31.8)	0.004
Calcium channel blocker	852 (55.9)	209 (54.9)	0.02
Cardiac glycoside	15 (1.0)	5 (1.3)	0.03
Loop diuretic	161 (10.6)	42 (11.0)	0.02
Other diuretic	168 (11.0)	41 (10.8)	0.01
Nitrate	248 (16.3)	67 (17.6)	0.04
Peripheral vasodilator	7 (0.5)	2 (0.5)	0.01
Platelet inhibitor	534 (35.0)	139 (36.5)	0.03
Respiratory			
Bronchodilator	1166 (76.5)	294 (77.2)	0.02
Inhaled corticosteroid	411 (27.0)	110 (28.9)	0.04
Psychotropic			
Antidepressant	191 (12.5)	49 (12.9)	0.01
Antipsychotic	81 (5.3)	20 (5.2)	0.003
Immune related			
Antibiotic	559 (36.7)	148 (38.8)	0.05
Immunosuppressant	5 (0.3)	1 (0.3)	0.01
Nonsteroidal or anti-inflammatory agent	381 (25.0)	94 (24.7)	0.01
Kidney			
Phosphate-binding agent	0	0	<0.001
Other			
Lipid-regulating agent	1244 (81.6)	295 (77.4)	0.10
Proton pump inhibitor	417 (27.4)	111 (29.1)	0.04
Systemic corticosteroid	231 (15.2)	57 (15.0)	0.01
Hormone replacement therapy	0	0	<0.001
Glucose-lowering agent			
Metformin	1305 (85.6)	331 (86.9)	0.04
Sulfonylurea	1100 (72.2)	274 (71.9)	0.01
Thiazolidinedione	148 (9.7)	41 (10.8)	0.04
Glucagon-like peptide-1 agonist	1 (0.1)	0	0.04
Acarbose	37 (2.4)	12 (3.1)	0.04
Meglitinide	0	0	<0.001
Insulin	235 (15.4)	57 (15.0)	0.01
**Diagnosis history (1 y prior)**			
Cardiovascular			
Coronary heart disease	192 (12.6)	48 (12.6)	<0.001
Heart failure	0	0	<0.001
Myocardial infarction	52 (3.4)	17 (4.5)	0.05
Cerebrovascular	66 (4.3)	17 (4.5)	0.01
Hypertensive disease	390 (25.6)	90 (23.6)	0.05
Arrhythmia or conduction disorder	51 (3.3)	14 (3.7)	0.02
Arterial disease	23 (1.5)	3 (0.8)	0.07
Kidney			
Chronic kidney disease	189 (12.4)	48 (12.6)	0.01
Respiratory			
Pneumonia	35 (2.3)	9 (2.4)	0.004
Acute bronchitis	35 (2.3)	7 (1.8)	0.03
Bronchiectasis	50 (3.3)	13 (3.4)	0.01
Extrinsic allergic alveolitis	0	0	<0.001
Other lung disease	236 (15.5)	54 (14.2)	0.04
Metabolic and endocrine			
Obesity	78 (5.1)	22 (5.8)	0.03
Hyperlipidemia	225 (14.8)	54 (14.2)	0.02
Thyroid disease	21 (1.4)	3 (0.8)	0.06
Osteoporosis	0	0	<0.001
Osteoporotic fracture	0	0	<0.001
All fractures	15 (1.0)	2 (0.5)	0.05
Paget disease	0	0	<0.001
Rheumatoid arthritis	0	0	<0.001
Cancer	51 (3.3)	13 (3.4)	0.004
Other			
Chronic pancreatitis	0	0	<0.001
Dementia	5 (0.3)	1 (0.3)	0.01
Liver disease (chronic liver disease, cirrhosis, esophageal varix, or hepatic failure)	12 (0.8)	2 (0.5)	0.03
Diabetes related			
Diabetic eye complication	93 (6.1)	22 (5.8)	0.01
Diabetic hyperosmolarity	0	0	<0.001
Diabetic neuropathy	22 (1.4)	9 (2.4)	0.07
Peripheral artery disease	6 (0.4)	2 (0.5)	0.02
Diabetic ketoacidosis	4 (0.3)	2 (0.5)	0.04
Biochemical parameter, mean (SD)			
Hemoglobin A_1c_, %	8.55 (1.69)	8.67 (1.46)	0.07
eGFR, mL/min/1.73 m^2^	85.76 (22.95)	85.94 (20.68)	0.01
Clinical history, mean (SD)			
No. of glucose-lowering agents used, 5 y prior^a^	1.93 (0.58)	1.97 (0.63)	0.06
Days since first diabetes diagnosis	3451 (1799)	3387 (1755)	0.04
No. of emergency department admissions, 1 y prior	0.31 (0.70)	0.35 (1.02)	0.04
No. of planned admissions, 1 y prior	0.37 (1.12)	0.36 (0.83)	0.01
Level of maintenance therapy for OAD	0.45 (0.70)	0.51 (0.74)	0.09
Comedication at baseline			
Metformin	1246 (81.8)	318 (83.5)	0.05
Sulfonylurea	946 (62.1)	239 (62.7)	0.01
Thiazolidinedione	106 (7.0)	28 (7.3)	0.02
Glucagon-like peptide-1 agonist	0	0	<0.001
Acarbose	24 (1.6)	9 (2.4)	0.06
Meglitinide	0	0	<0.001
Insulin	542 (35.6)	135 (35.4)	0.003
Total, mean (SD)	1.88 (0.71)	1.91 (0.74)	0.05

Abbreviations: DPP4I, dipeptidyl peptidase-4 inhibitor; eGFR, estimated glomerular filtration rate; OAD, obstructive airway disease; SGLT2I, sodium-glucose cotransporter 2 inhibitor; SMD, standardized mean difference.

^a^ Metformin, sulfonylureas, meglitinides, glucagon-like peptide receptor agonists, acarbose, and thiazolidinediones.

### Association of SGLT2I Use With Incident OAD

The non-OAD cohort was followed for a median (IQR) of 2.3 (1.0-3.5) years for the DPP4I group and 1.2 (0.5-3.0) years for the SGLT2I group. Compared with DPP4I use, SGLT2I use was associated with a 35% reduced risk of incident OAD (HR, 0.65 [95% CI, 0.54-0.79]; *P* < .001) (Table 3 and Kaplan-Meier plot in eFigure 2 in Supplement 1). The 1-year ARD for incident OAD was 0.66% and the NNT was 152. The association remained in sensitivity analyses that adopted more stringent OAD definitions (eTable 3 in Supplement 1).

**Table 3.** Association of Sodium-Glucose Cotransporter 2 Inhibitors With Risk of Incident Obstructive Airway Disease

Group	No. of patients	No. of events	Total person-years	Median follow-up (IQR), y	Hazard ratio (95% CI)	*P* value
DPP4I	22 784	1069	53 407	2.3 (1.0-3.5)	1 [Reference]	NA
SGLT2I	5696	135	10 478	1.2 (0.5-3.0)	0.65 (0.54-0.79)	<.001

Abbreviations: DPP4I, dipeptidyl peptidase-4 inhibitor; NA, not applicable; SGLT2I, sodium-glucose cotransporter 2 inhibitor.

In the subgroup analysis by sex, associations were observed among men (HR, 0.66 [95% CI, 0.51-0.86]; *P* = .002) and among women (HR, 0.73 [95% CI, 0.54-0.97]; *P* = .03) (Table 4 and Kaplan-Meier plots in eFigures 3 and 4 in Supplement 1, respectively). There was no evidence of interaction (*P* for interaction = .52). In men, the 1-year ARD was 0.62% and the NNT was 161; in women, the 1-year ARD was 0.54% and the NNT was 186. In men, the association remained in sensitivity analyses that adopted more stringent OAD definitions (eTable 4 in Supplement 1). In women, although the effect estimates were similar, there were no associations in the sensitivity analyses (eTable 5 in Supplement 1).

**Table 4.** Subgroup Analysis (by Sex) for the Association of Sodium-Glucose Cotransporter 2 Inhibitors With Risk of Incident Obstructive Airway Disease

Group	No. of patients	No. of events	Total person-years	Median follow-up (IQR), y	Hazard ratio (95% CI)	*P* value^a^
Men						
DPP4I	12 404	586	28 896	2.3 (1.0-3.5)	1 [Reference]	NA
SGLT2I	3101	74	5910	1.4 (0.5-3.1)	0.66 (0.51-0.86)	.002
Women						
DPP4I	9360	485	22 157	2.4 (1.1-3.5)	1 [Reference]	NA
SGLT2I	2340	59	4048	1.1 (0.4-2.8)	0.73 (0.54-0.97)	.03

Abbreviations: DPP4I, dipeptidyl peptidase-4 inhibitor; NA, not applicable; SGLT2I, sodium-glucose cotransporter 2 inhibitor.

^a^ *P* for interaction = .52.

### Association of SGLT2I Use With OAD Exacerbation Events

The OAD cohort was followed for a median (IQR) of 2.3 (1.0-3.5) years in the DPP4I group and 1.5 (0.5-3.0) years in the SGLT2I group. The Poisson (count) model showed that compared with DPP4I use, SGLT2I use was associated with a 46% reduced rate of OAD exacerbations (RR, 0.54 [95% CI, 0.36-0.83]; *P* = .01; Table 5). The zero model showed no evidence of a difference in the chance of having excess zero counts between the 2 exposure groups (*P* = .78). The association remained in sensitivity analyses (eTable 6 in Supplement 1).

**Table 5.** Association of Sodium-Glucose Cotransporter 2 Inhibitors With Rate of Obstructive Airway Disease Exacerbation Events

Group	No. of patients	No. of events	Total person-years	Median follow-up (IQR), y	Count model		Zero model	
					Rate ratio (95% CI)	*P* value	Odds ratio (95% CI)	*P* value
DPP4I	1524	526	3575	2.3 (1.0-3.5)	1 [Reference]	NA	1 [Reference]	NA
SGLT2I	381	54	732	1.5 (0.5-3.0)	0.54 (0.36-0.83)	.01	1.08 (0.65-1.80)	.78

Abbreviations: DPP4I, dipeptidyl peptidase-4 inhibitor; NA, not applicable; SGLT2I, sodium-glucose cotransporter 2 inhibitor.

## Discussion

This cohort study investigated the association of SGLT2I use with OAD incidence and exacerbation using a retrospective cohort with 30 385 patients with type 2 diabetes from routine clinical practice in Hong Kong, who were matched using a prevalent new-user design and PS matching. We observed that compared with DPP4I use, SGLT2I use was associated with a reduced risk of incident OAD and a lower rate of OAD exacerbations. Our results from the zero model suggested that there was no differential association with excess zero exacerbation events between SGLT2I and DPP4I use. Sex subgroup analysis for incident showed consistent results.

Clinical studies on the association of SGLT2I use with OAD are lacking. In a recent population-based study comparing the risk of asthma exacerbations between glucagon-like peptide 1 (GLP-1) agonists and other antidiabetic agents, SGLT2I use was associated with a higher rate of exacerbations.^23^ However, no direct comparison between SGLT2Is and DPP4Is was made.^23^ This study provided a clinical comparison of the association of OAD with SGLT2Is and DPP4Is. One interpretation of these results is that rather than SGLT2Is being protective against OAD, DPP4Is may be detrimental. However, published studies showed no associations of DPP4I use with OAD outcomes.^27,28^ More importantly, our results were in agreement with a recent meta-analysis^19^ and a network meta-analysis^20^ of cardiorenal randomized clinical trials showing that SGLT2I use was associated with a lower risk of OAD adverse events compared with placebo or DPP4I use.

One strength of this study was the use of the prevalent new-user design.^24^ Since DPP4Is are introduced before SGLT2Is, it is common for a patient to have previous exposure to DPP4Is before the initiation of SGLT2Is. The traditional new-user design would exclude such patients. The prevalent new-user design used here allows these patients in the cohort by matching the length of previous exposure to DPP4Is, which would better reflect clinical practice. To account for potential bias due to excess zero exacerbation counts, the ZIP regression model was used to estimate the rate of OAD exacerbations. Furthermore, the use of metformin and GLP-1 agonists, which have been shown to associate with improved asthma control,^23,29,30^ was included in PS matching together with other important covariates.

Multiple sensitivity analyses were performed to assess the results under more stringent definitions of OAD. In Hong Kong, short-acting bronchodilators may have uncommon single use in relieving acute bronchitis. Therefore, in the incident OAD sensitivity analysis, the restriction of 7 days or longer on the prescription of short-acting bronchodilators would exclude those prescriptions indicated for acute bronchitis. The other sensitivity analysis, which excluded any outcome events when patients were diagnosed with acute bronchitis or heart failure at any time during follow-up, would exclude potential misdiagnosis of OAD due to acute bronchitis or heart failure symptoms. In the exacerbation analysis, systemic corticosteroids were restricted to short-course oral corticosteroids from emergency department or inpatient visits only to minimize the possibility that corticosteroids were indicated for conditions other than exacerbations.^21,22^ Clinical guidelines^31,32,33^ also recommend the use of short-course oral corticosteroids for exacerbations unless patients are unable to tolerate the oral route. To capture these patients, a prescription of injected corticosteroids from an emergency department or inpatient visit was added to the definition of OAD exacerbations in the sensitivity analysis. Furthermore, heart failure symptoms may be misdiagnosed as OAD exacerbations. Therefore, we conducted another sensitivity analysis that excluded any exacerbation events when patients were diagnosed with heart failure at any time during follow-up; our results suggest an association against potential misdiagnosis of OAD exacerbations.

The mechanism underlying the association of SGLT2I use with risk of OAD and exacerbations is unclear. One possible mechanism may be related to the potential anti-inflammatory property of SGLT2Is. Mouse studies showed that SGLT2Is reduced proinflammatory markers in kidney cells^34^ and liver cells.^14^ A human cohort study also reported that SGLT2Is reduced proinflammatory markers and increased anti-inflammatory cytokine in patients with type 2 diabetes.^35^ Furthermore, SGLT2Is were shown to inhibit NLRP3 inflammasome activation in multiple tissues, including the heart,^13^ liver,^14^ kidney,^15^ and lung.^16^ To our knowledge, no experimental studies have directly evaluated the association of SGLT2I use with OAD; however, NLRP3 inflammasome activation has been implicated in asthmatic airway inflammation^17^ and COPD exacerbations.^18^ Therefore, SGLT2Is may potentially alleviate OAD symptoms through inhibition of the NLRP3 inflammasome.

This study has important clinical implications. Type 2 diabetes is associated with increased risk of OAD and worsened control. Therefore, the identification of antidiabetic agents that harbor protective effects against OAD is of clinical importance. Although the results from a single observational study cannot provide conclusive evidence to inform clinical practice, this study provides observational evidence that SGLT2I use may harbor additional protective effects against OAD in patients with type 2 diabetes. The effects of SGLT2Is on the cardiorenal systems have been studied extensively, while the role of SGLT2Is in the respiratory system remains largely unexplored. This study raises the possibility that SGLT2Is may play an important role in the treatment of chronic respiratory diseases, such as COPD and asthma, and that further investigation is warranted.

### Limitations

This study has some limitations. First, the CDARS database does not contain complete records on smoking status. However, it is unlikely that smoking would confound the decision to prescribe either SGLT2Is or DPP4Is. Furthermore, to account for the difference in preindex OAD severity, the PS model included the preindex levels of OAD maintenance therapy (eTable 2 in Supplement 1). Second, although the dispensing records in the CDARS database are highly complete, the clinical diagnosis may be underreported, which is an inherent limitation of many electronic health care databases. Therefore, we did not use a diagnosis plus a prescription in defining OAD outcomes to prevent low sensitivity. Third, there was a potential by-indication bias. The need to add or switch to SGLT2Is from DPP4Is may be due to poor glycemic control or the presence of comorbidities, which would result in worse disease outcomes among SGLT2I users. However, if such bias existed, it would only underestimate the beneficial effects of SGLT2Is without affecting the overall conclusion. Fourth, in the subgroup analyses, the association with incident OAD in women was attenuated in the sensitivity analyses. Since the estimates remained similar to those of the main analysis, the nonsignificant results may be due to the reduced sample size and event number in the subgroup. Fifth, it is unknown whether SGLT2Is can change the pathogenesis of OAD. Ascertainment of the “incident” events is limited by the clinical presentations of OAD, meaning that SGLT2Is may reduce OAD symptoms rather than prevent a patient from developing the disease.

## Conclusions

In this retrospective cohort study of patients with type 2 diabetes in Hong Kong, SGLT2I use was associated with a reduced risk of incident OAD as well as a lower rate of OAD exacerbations in clinical settings compared with DPP4I use. Similar results were also observed among men and women.

## Notes

Supplement 1.
eMethods.
**eFigure 1.** Study Flow Diagram **eFigure 2.** Kaplan-Meier Plot for Incident Obstructive Airway Disease in the Main Analysis **eFigure 3.** Kaplan-Meier Plot for Incident Obstructive Airway Disease in Men **eFigure 4.** Kaplan-Meier Plot for Incident Obstructive Airway Disease in Women **eTable 1.** List of Conditions in the Exclusion Criteria **eTable 2.** List of Covariates Included for Propensity Score Calculation **eTable 3.** Sensitivity Analysis of the Association of Sodium-Glucose Cotransporter 2 Inhibitors With Risk of Incident Obstructive Airway Disease **eTable 4.** Sensitivity Analysis of the Association of Sodium-Glucose Cotransporter 2 Inhibitors With Risk of Incident Obstructive Airway Disease in Men **eTable 5.** Sensitivity Analysis of the Association of Sodium-Glucose Cotransporter 2 Inhibitors With Risk of Incident Obstructive Airway Disease in Women **eTable 6.** Sensitivity Analysis of the Association of Sodium-Glucose Cotransporter 2 Inhibitors With the Rate of Obstructive Airway Disease Exacerbation Events
eReferences

Supplement 2.
Data Sharing Statement
