# Supplementary material for: Association of Sodium-Glucose Cotransporter 2 Inhibitor vs Dipeptidyl Peptidase-4 Inhibitor Use With Risk of Incident Obstructive Airway Disease and Exacerbation Events Among Patients With Type 2 Diabetes in Hong Kong
Source: JAMA Netw Open. 2023 Jan 17;6(1):e2251177. doi: 10.1001/jamanetworkopen.2022.51177 (PMC9857182; doi:10.1001/jamanetworkopen.2022.51177)
Supplement: Supplement 1. — eMethods. eFigure 1. Study Flow Diagram eFigure 2. Kaplan-Meier Plot for Incident Obstructive Airway Disease in the Main Analysis eFigure 3. Kaplan-Meier Plot for Incident Obstructive Airway Disease in Men eFigure 4. Kaplan-Meier Plot for Incident Obstructive Airway Disease in Women eTable 1. List of Conditions in the Exclusion Criteria eTable 2. List of Covariates Included for Propensity Score Calculation eTable 3. Sensitivity Analysis of the Association of Sodium-Glucose Cotransporter 2 Inhibitors With Risk of Incident Obstructive Airway Disease eTable 4. Sensitivity Analysis of the Association of Sodium-Glucose Cotransporter 2 Inhibitors With Risk of Incident Obstructive Airway Disease in Men eTable 5. Sensitivity Analysis of the Association of Sodium-Glucose Cotransporter 2 Inhibitors With Risk of Incident Obstructive Airway Disease in Women eTable 6. Sensitivity Analysis of the Association of Sodium-Glucose Cotransporter 2 Inhibitors With the Rate of Obstructive Airway Disease Exacerbation Events eReferences [file jamanetwopen-e2251177-s001.pdf]

Supplementary Online Content

Au PCM, Tan KCB, Lam DCL, et al. Association of sodium-glucose cotransporter 2 inhibitor vs dipeptidyl peptidase-4 inhibitor use with risk of incident obstructive airway disease and exacerbation events among patients with type 2 diabetes in Hong Kong. *JAMA Netw Open*. 2023;6(1):e2251177. doi:10.1001/jamanetworkopen.2022.51177

eMethods.
eFigure 1. Study Flow Diagram
eFigure 2. Kaplan-Meier Plot for Incident Obstructive Airway Disease in the Main Analysis
eFigure 3. Kaplan-Meier Plot for Incident Obstructive Airway Disease in Men
eFigure 4. Kaplan-Meier Plot for Incident Obstructive Airway Disease in Women
eTable 1. List of Conditions in the Exclusion Criteria
eTable 2. List of Covariates Included for Propensity Score Calculation
eTable 3. Sensitivity Analysis of the Association of Sodium-Glucose Cotransporter 2 Inhibitors With Risk of Incident Obstructive Airway Disease
eTable 4. Sensitivity Analysis of the Association of Sodium-Glucose Cotransporter 2 Inhibitors With Risk of Incident Obstructive Airway Disease in Men
eTable 5. Sensitivity Analysis of the Association of Sodium-Glucose Cotransporter 2 Inhibitors With Risk of Incident Obstructive Airway Disease in Women
eTable 6. Sensitivity Analysis of the Association of Sodium-Glucose Cotransporter 2 Inhibitors With the Rate of Obstructive Airway Disease Exacerbation Events
eReferences

This supplementary material has been provided by the authors to give readers additional information about their work.

© 2023 Au PCM et al. *JAMA Network Open.*

eMethods

Data Source

The Clinical Data Analysis and Reporting System (CDARS) is a territory-wide representative electronic medical database from the Hospital Authority (HA) of Hong Kong. The HA manages all 42 public hospitals and 120 public outpatient general and specialist clinics in Hong Kong. More than 90% of the known patients with diabetes in Hong Kong are under the HA's care[1]. The CDARS is an ethnically homogeneous population of about 92% Han Chinese[2]. It contains clinical records from outpatient, emergency, and inpatient visits, including diagnosis, dispensing, clinical procedures and operations, laboratory tests, and death registry records. Since CDARS is a territory-wide database covering all public hospitals and outpatient clinics in Hong Kong, lost to follow-up would be unlikely.

Exclusion Criteria

The exclusion criteria were: 1) patients in the DPP4i control group with any uses of SGLT2is before index date; 2) patients with DPP4i and SGLT2i first initiated on the same date; 3) patients of type 1 diabetes; 4) patients with prescription records of index drugs for only one day; 5) patients with diseases routinely treated with systemic corticosteroids[3], or with diseases affecting the respiratory system, except for OAD[3] (eTable 1); 6) patients with heart failure; 7) patients who received dialysis, kidney transplant, or other tissue and organ transplant within one year before index date; and 8) patients with no laboratory measurements of HbA1c or eGFR within one year before index date. Type 1 diabetes was defined according to a previous validation study[4]: 1) the number of type 1 diabetes diagnosis records to the number of type 2 diabetes diagnosis records ratio ≥4[4]; 2) prescribed with insulin and no other glucose-lowering agents within the first year of diabetes diagnosis[4]; or 3) age at diagnosis <30. eGFR was estimated using the new Asian modified CKD-EPI equation[5].

"Prevalent New-User" Design

The design matched study participants based on the length of previous exposure to DPP4is in a time-dependent manner[6]. The patients in the SGLT2i group were patients who were new users of SGLT2is or ongoing users of DPP4is who switched to/added SGLT2is. The "prevalent new-user" design matched these SGLT2i users with ongoing users of DPP4is (but have not switched to SGLT2is) based on their length of previous use of DPP4is in a time-dependent manner, meaning that a DPP4i user could be matched with multiple SGLT2i users at different time-points over the course of DPP4i use. The baseline characteristics of the matched pairs were then assessed for propensity score (PS) calculation. Unlike the conventional new-user design, it allowed SGLT2is users with previous or ongoing use of DPP4is to be included in the cohort. For patients first initiating SGLT2is without previous use of DPP4is (i.e. new users), they were matched with patients first initiating DPP4is. The "prevalent new-user" design allowed an unbiased comparison between patients who switched to or added SGLT2is from DPP4is and patients who stayed on DPP4is.

Propensity Score (PS) Calculation and Matching

PS was calculated using conditional logistic regression stratified by the pairs matched in the "prevalent new-user" design. PS matching was done within each "prevalent new-user" matched pair using sequential greedy matching[7] with a calliper of 0.2 standard deviations (SD). The patients were matched 1:4 (SGLT2is:DPP4is) without replacement within and across "prevalent new-user" matched pairs.

© 2023 Au PCM et al. *JAMA Network Open.*

eFigure 1. Study Flow Diagram

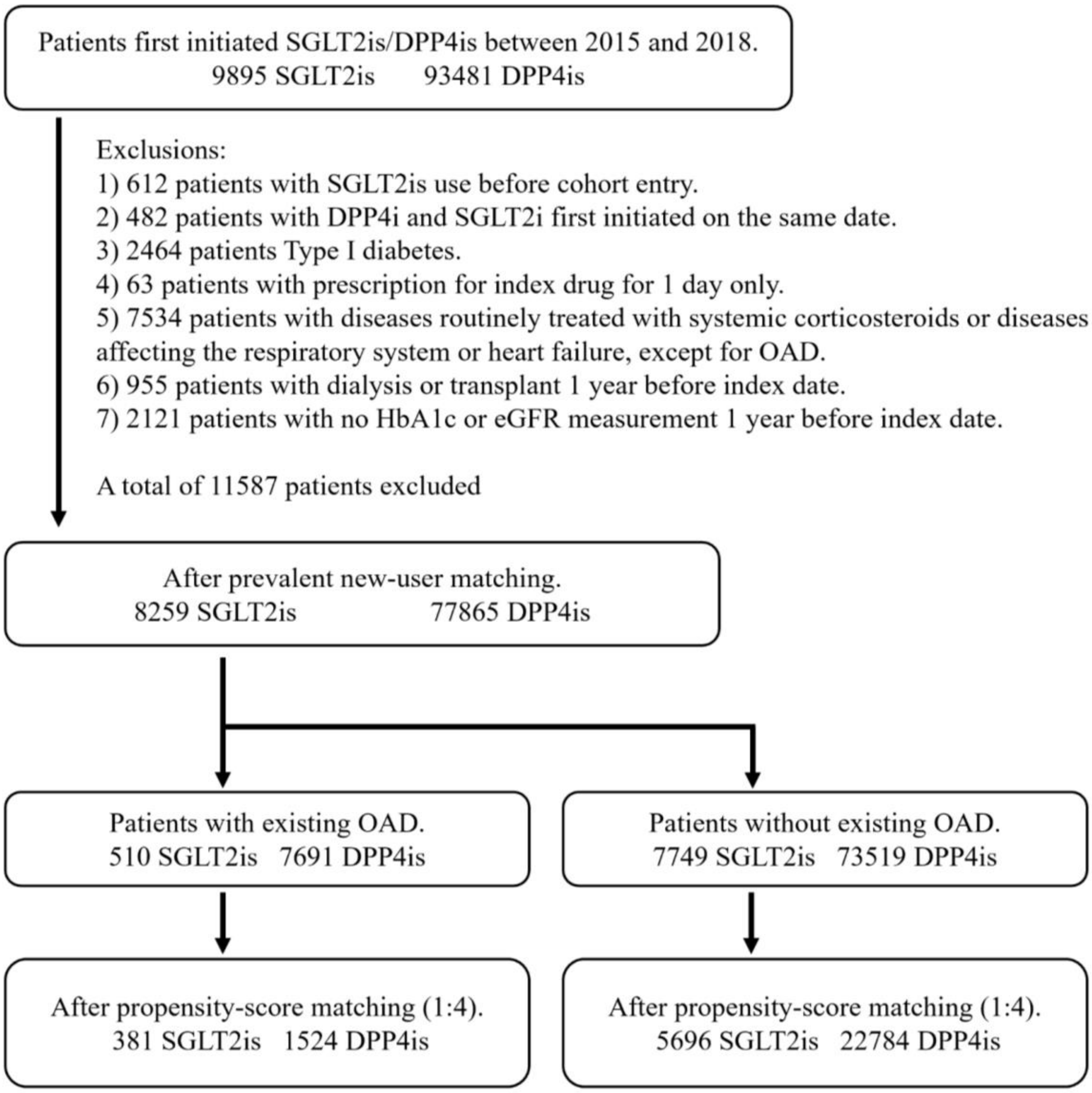

© 2023 Au PCM et al. *JAMA Network Open.*

eFigure 2. Kaplan-Meier Plot for Incident Obstructive Airway Disease in the Main Analysis

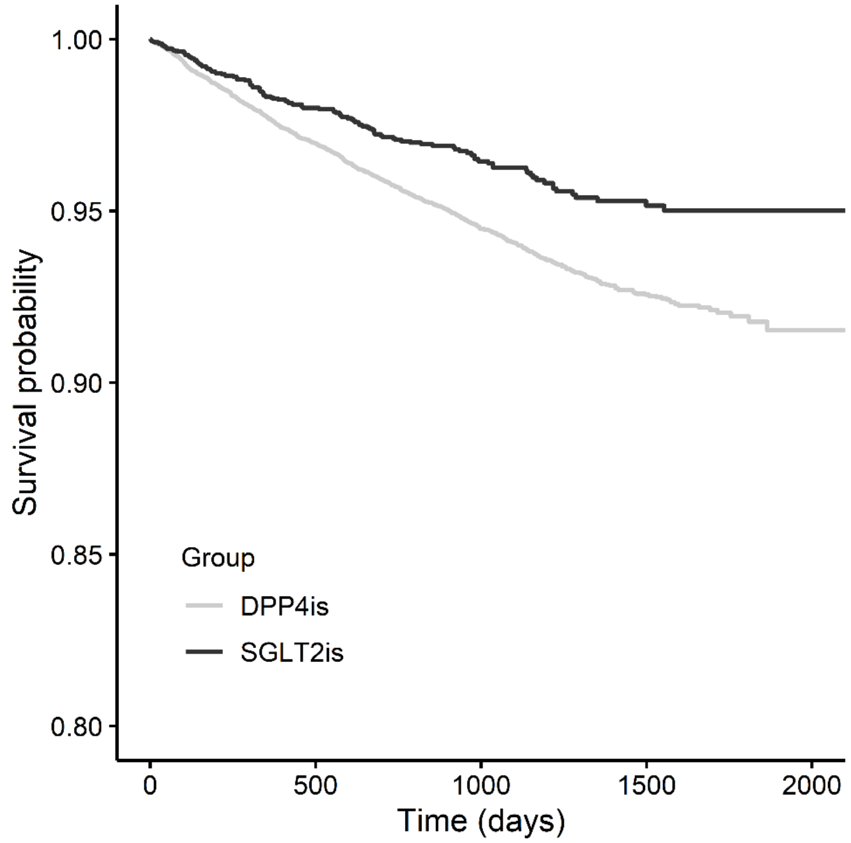

© 2023 Au PCM et al. *JAMA Network Open.*

eFigure 3. Kaplan-Meier Plot for Incident Obstructive Airway Disease in Men

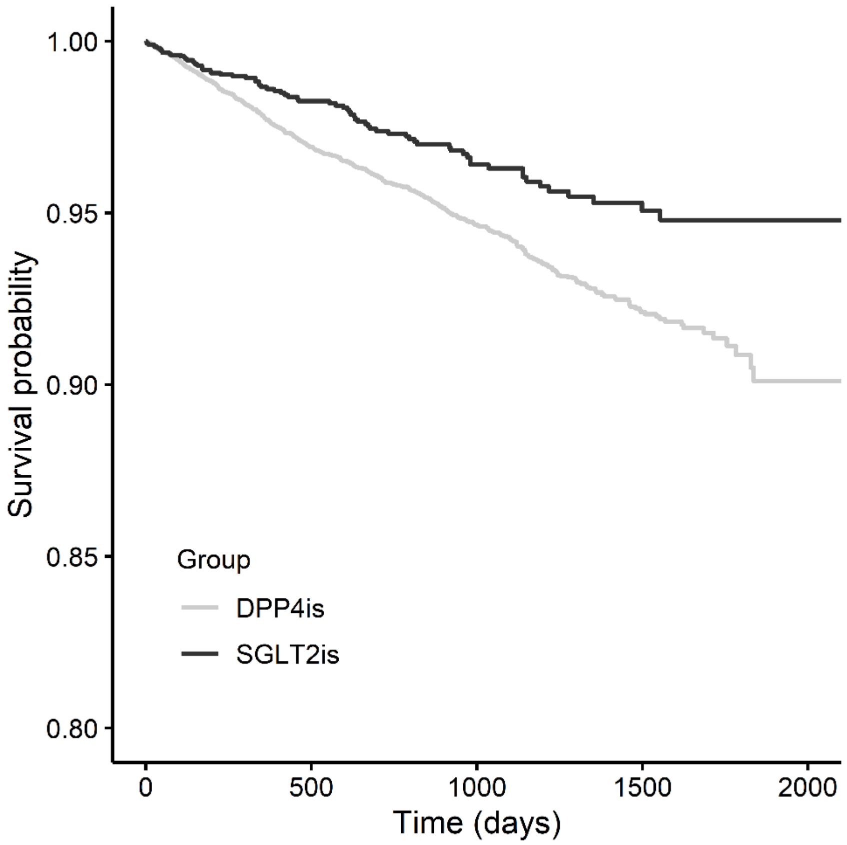

© 2023 Au PCM et al. *JAMA Network Open.*

eFigure 4. Kaplan-Meier Plot for Incident Obstructive Airway Disease in Women

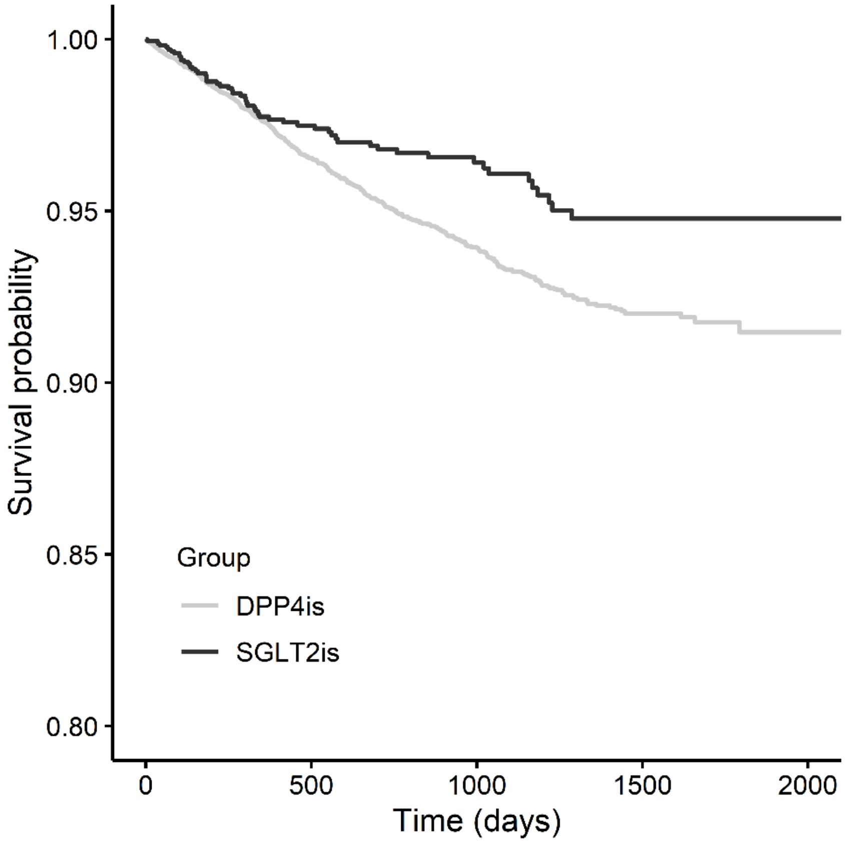

© 2023 Au PCM et al. *JAMA Network Open.*

eTable 1. List of Conditions in the Exclusion Criteria

**Diseases affecting the respiratory system, except for OAD**	**ICD-9-CM Definitions**
Allergic bronchopulmonary aspergillosis	518.6
Alpha 1-antitrypsin deficiency	273.4
Aspiration syndromes	934.8
Benign neoplasms of bronchus or lung	212.3
Bronchopulmonary dysplasia	770.7
Cancers of lymphatic and hematopoietic systems	200, 201, 202, 203, 204, 205, 206, 207, 208
Cerebral palsy	343
Congenital airway and lung abnormalities	748.2, 748.3, 748.4, 748.5, 748.6, 748.7, 748.8, 748.9
Chronic pulmonary heart diseases	416
Heart failure	398.91, 402.01, 402.11, 402.91, 404.01, 404.11, 404.91, 428
Cystic fibrosis and metabolic disorders	277
Disorders of diaphragm	519.4
Lung involvement in conditions classified elsewhere	517
Motor neuron diseases	335
Myopathies	359
Pneumoconioses	500, 501, 502, 503, 504, 505, 506, 507, 508
Primary and secondary malignant neoplasm of airway, bronchus, lung or respiratory organs	160, 161, 162, 163, 164, 165, 197.0, 197.1, 197.2, 197.3
Pulmonary alveolar and parietoalveolar pneumonopathies	516
Pulmonary fibrosis	515
Vocal cord and laryngeal disease	478.3, 478.5
**Diseases routinely treated with systemic corticosteroids**	
Inflammatory arthropathies	714
Post-transplant lymphoproliferative disorder	238.77
Systemic lupus erythematosus, scleroderma, systemic sclerosis, dermatomyositis	710
Organ or tissue transplant	V42, V49.83, V58.44, E878.0, 996.8 or<br>ICD-9 Procedure codes: 55.69, 41.00, 50.59, 37.59, 33.59, 41.04, 41.03, 50.51, 33.51

© 2023 Au PCM et al. *JAMA Network Open.*

eTable 2. List of Covariates Included for Propensity Score Calculation

Covariates	Definitions
Sex	---
Age at index date	---
Season of index date	Winter (Dec - Feb), Spring (Mar - May), Summer (Jun - Aug), Fall (Sept - Nov)
**Medication History (1 year prior)**	
**Cardiovascular**	**B.N.F.**
Angiotensin-converting-enzyme inhibitors / angiotensin II receptor blockers	2.5.5.1, 2.5.5.2, 2.5.5.3
Antiarrhythmic agents	2.3.2
Anticoagulants	2.8
Beta blockers	2.4
Calcium channel blockers	2.6.2
Cardiac glycosides	2.1.1
Loop diuretics	2.2.2
Other diuretics	2.2.1, 2.2.3, 2.2.4, 2.2.5
Nitrates	2.6.1
Peripheral vasodilators	2.6.4
Platelet inhibitors	2.9
**Respiratory**	**B.N.F.**
Bronchodilators	3.1
Inhaled corticosteroids (Including corticosteroids / bronchodilators / long-acting beta-agonists combinations)	3.2
**Immune related**	**B.N.F.**
Antibiotics	5.1
Immunosuppressants	8.2
Non-steroidal and anti-inflammatory	10.1.1
**Psychotropic**	**B.N.F.**
Antidepressants	4.3
Antipsychotics	4.2
**Renal**	**B.N.F.**
Phosphate binding agents	9.5.2.2
**Others**	**B.N.F.**
Lipid regulating agents	2.12
Proton pump inhibitors	1.3.5
Systemic corticosteroids	6.3
Hormone replacement therapy	6.4.1.1

© 2023 Au PCM et al. *JAMA Network Open.*

Medication History (1 year prior)	
**Glucose-lowering agents**	
Metformin	---
Sulfonylureas	---
Thiazolidinediones	---
Glucagon-like peptide-1 agonists	---
Acarbose	---
Insulin	---
**Diagnosis History (1 year prior)**	
**Cardiovascular**	**ICD-9-CM**
Coronary heart disease	410, 411, 412, 413, 414, 429.2, 429.71, 429.79
Myocardial infarction	410, 412
Cerebrovascular	362.34, 430, 431, 432, 433, 434, 435, 436, 437, 438
Hypertensive disease	401, 402, 403, 404, 405
Arrhythmia and conduction disorders	426, 427
Arterial disease	433.00, 433$, 433.10, 433.20, 433.30, 433.80, 433.90, 440, 441, 442, 443, 444, 445, 447, 250.7
**Renal**	
Chronic renal diseases	Latest eGFR measurement <60 mL/min/1.73m^2
**Respiratory**	**ICD-9-CM**
Pneumonia	481, 482, 483, 485, 486
Acute bronchitis	466, 490
Bronchiectasis	494
Extrinsic allergic alveolitis	495
Other lung diseases	460, 461, 462, 463, 464, 465, 466, 470, 472, 473, 474, 475, 476, 477, 478, 510, 511, 512, 513, 514, 515, 516, 517, 518, 519
**Metabolic and endocrine**	**ICD-9-CM**
Obesity	278.0, 278
Hyperlipidaemia	272.0, 272.1, 272.2, 272.4
Thyroid disease	242, 243, 244
Osteoporosis	733.0
Osteoporotic fractures	820, 805, 812, 813, 814
All fractures	800 - 829
Paget's diseases	731.0
Rheumatoid arthritis	710.0, 710.1, 710.4, 714.0, 714.1, 714.2, 714.81, 725
Cancers	140 - 208
**Others**	**ICD-9-CM**
Chronic pancreatitis	577.1
Dementia	290

© 2023 Au PCM et al. *JAMA Network Open.*

**Diagnosis History (1 year prior)**	
Liver diseases (chronic liver disease, cirrhosis, esophageal varices, and hepatic failure)	456.0, 456.1, 456.2, 571.2, 571.4, 571.5, 571.6, 572.2, 572.3, 572.4, 572.8
**Diabetes related**	**ICD-9-CM**
Diabetic eye complications	366.41, 362.0, 250.5
Diabetic hyperosmolarity	250.2
Diabetic neuropathy	250.6
Peripheral artery diseases	440.2, 440.4, 443.9, 249.70, 249.71, 250.70, 250.71, 250.72, 250.73
Diabetic Ketoacidosis	250.1, 250.3
**Biochemical parameters (latest measurement within 1 year)**	
HbA1c % / mmol/mol	---
eGFR (mL/min/1.73m^2)	The new Asian modified CKD-EPI equation[5]
**Clinical History**	
No. of glucose-lowering agents used,<br>5 years prior	Metformin, Sulfonylureas, Meglitinides, GLP-1 receptor agonists, Acarbose, Thiazolidinediones
Days since first diabetes diagnosis	---
No. of emergency admissions,<br>1 year prior	---
No. of planned admissions,<br>1 year prior	---
Level of maintenance therapy for OAD	Level 1: none<br>Level 2: inhaled corticosteroids only or long-acting bronchodilators only<br>Level 3: inhaled corticosteroids + long-acting bronchodilators
**Co-medication at baseline**	
Metformin	---
Sulfonylureas	---
Thiazolidinediones	---
Glucagon-like peptide-1 agonists	---
Acarbose	---
Insulin	---
Total number of the above co-medications	---

© 2023 Au PCM et al. *JAMA Network Open.*

eTable 3. Sensitivity Analysis of the Association of Sodium-Glucose Cotransporter 2 Inhibitors With Risk of Incident Obstructive Airway Disease

Group	No. of subjects	No. of events	Total person-year	Median follow-up in year (IQR)	Hazard ratio (95% CI)	P
OAD diagnosis or ICS or LTRA or PDE4 inhibitors or LABD or at least 7 days of SABD						
DPP4i	22784	686	54136	2.4 (1.1-3.5)	1	---
SGLT2i	5696	74	10578	1.3 (0.5-3.0)	0.59 (0.46-0.76)	<0.001
OAD diagnosis or ICS or LTRA or PDE4 inhibitors or LABD or SABD (excluded events with a diagnosis of acute bronchitis or heart failure anytime during follow-up)						
DPP4i	22784	881	53657	2.3 (1.1-3.5)	1	---
SGLT2i	5696	120	10491	1.2 (0.5-3.0)	0.68 (0.56-0.84)	<0.001

ICS, inhaled corticosteroids; LTRA, leukotriene receptor antagonists; PDE4, phosphodiesterase-4; LABD, long-acting bronchodilators; SABD, short-acting bronchodilators.

© 2023 Au PCM et al. *JAMA Network Open.*

eTable 4. Sensitivity Analysis of the Association of Sodium-Glucose Cotransporter 2 Inhibitors With Risk of Incident Obstructive Airway Disease in Men

Group	No. of subjects	No. of events	Total person-year	Median follow-up in year (IQR)	Hazard ratio (95% CI)	P
OAD diagnosis or ICS or LTRA or PDE4 inhibitors or LABD or at least 7 days of SABD						
DPP4i	12404	406	29202	2.4 (1.1-3.5)	1	---
SGLT2i	3101	40	5966	1.4 (0.5-3.1)	0.50 (0.35-0.71)	<0.001
OAD diagnosis or ICS or LTRA or PDE4 inhibitors or LABD or SABD (excluded events with a diagnosis of acute bronchitis or heart failure anytime during follow-up)						
DPP4i	12404	485	29000	2.3 (1.1-3.5)	1	---
SGLT2i	3101	66	5915	1.4 (0.5-3.1)	0.74 (0.56-0.97)	0.03

ICS, inhaled corticosteroids; LTRA, leukotriene receptor antagonists; PDE4, phosphodiesterase-4; LABD, long-acting bronchodilators; SABD, short-acting bronchodilators.

eTable 5. Sensitivity Analysis of the Association of Sodium-Glucose Cotransporter 2 Inhibitors With Risk of Incident Obstructive Airway Disease in Women

Group	No. of subjects	No. of events	Total person-year	Median follow-up in year (IQR)	Hazard ratio (95% CI)	P
OAD diagnosis or ICS or LTRA or PDE4 inhibitors or LABD or at least 7 days of SABD						
DPP4i	9360	286	22543	2.4 (1.1-3.6)	1	---
SGLT2i	2340	34	4088	1.1 (0.4-2.8)	0.74 (0.50-1.08)	0.12
OAD diagnosis or ICS or LTRA or PDE4 inhibitors or LABD or SABD (excluded events with a diagnosis of acute bronchitis or heart failure anytime during follow-up)						
DPP4i	9360	392	22289	2.4 (1.1-3.6)	1	---
SGLT2i	2340	51	4057	1.1 (0.4-2.8)	0.78 (0.57-1.06)	0.11

ICS, inhaled corticosteroids; LTRA, leukotriene receptor antagonists; PDE4, phosphodiesterase-4; LABD, long-acting bronchodilators; SABD, short-acting bronchodilators.

eTable 6. Sensitivity Analysis of the Association of Sodium-Glucose Cotransporter 2 Inhibitors With the Rate of Obstructive Airway Disease Exacerbation Events

					Count model			Zero model	
Group	No. of subjects	No. of events	Total person-year	Median follow-up in year (IQR)	Rate ratio (95% CI)	P		Odd ratio (95% CI)	P
OAD diagnosis from IP or AE, or short-course OCS, or injected CS									
DPP4i	1524	539	3575	2.3 (1.0-3.5)	1	---		1	---
SGLT2i	381	54	732	1.5 (0.5-3.0)	0.51 (0.33-0.79)	0.003		1.02 (0.61-1.73)	0.93
OAD diagnosis from IP or AE, or short-course OCS (excluded events with a diagnosis of heart failure anytime during follow-up)									
DPP4i	1524	429	3574	2.3 (1.0-3.5)	1	---		1	---
SGLT2i	381	48	732	1.5 (0.5-3.0)	0.51 (0.33-0.81)	0.004		0.95 (0.54-1.67)	0.87

IP, inpatient; AE, accident and emergency; OCS, oral corticosteroids; CS corticosteroids.

© 2023 Au PCM et al. *JAMA Network Open.*

eReferences

1. Lau IT. A Clinical Practice Guideline to Guide a System Approach to Diabetes Care in Hong Kong. *Diabetes Metab J*. Apr 2017;41(2):81-88. doi:10.4093/dmj.2017.41.2.81
2. The Census and Statistics Department tGotHKSAR. Hong Kong 2016 Population By-census - Thematic Report : Ethnic Minorities. Updated 29 Dec 2017. Accessed 11 Oct 2021, https://www.censtatd.gov.hk/en/EIndexbySubject.html?pcode=B1120100&scode=459
3. Foer D, Beeler PE, Cui J, Karlson EW, Bates DW, Cahill KN. Asthma Exacerbations in Patients with Type 2 Diabetes and Asthma on Glucagon-like Peptide-1 Receptor Agonists. *Am J Respir Crit Care Med*. Apr 1 2021;203(7):831-840. doi:10.1164/rccm.202004-0993OC
4. Luk AOY, Ke C, Lau ESH, et al. Secular trends in incidence of type 1 and type 2 diabetes in Hong Kong: A retrospective cohort study. *PLoS Med*. Feb 2020;17(2):e1003052. doi:10.1371/journal.pmed.1003052
5. Wang J, Xie P, Huang JM, et al. The new Asian modified CKD-EPI equation leads to more accurate GFR estimation in Chinese patients with CKD. *Int Urol Nephrol*. Dec 2016;48(12):2077-2081. doi:10.1007/s11255-016-1386-9
6. Suissa S, Moodie EE, Dell'Aniello S. Prevalent new-user cohort designs for comparative drug effect studies by time-conditional propensity scores. *Pharmacoepidemiol Drug Saf*. Apr 2017;26(4):459-468. doi:10.1002/pds.4107
7. Austin PC. A comparison of 12 algorithms for matching on the propensity score. *Stat Med*. Mar 15 2014;33(6):1057-69. doi:10.1002/sim.6004

© 2023 Au PCM et al. *JAMA Network Open.*
