# Supplementary material for: Association of Sodium-Glucose Cotransporter 2 Inhibitor vs Dipeptidyl Peptidase-4 Inhibitor Use With Risk of Incident Obstructive Airway Disease and Exacerbation Events Among Patients With Type 2 Diabetes in Hong Kong
Source: JAMA Netw Open. 2023 Jan 17;6(1):e2251177. doi: 10.1001/jamanetworkopen.2022.51177 (PMC9857182; doi:10.1001/jamanetworkopen.2022.51177)
Supplement: Supplement 2. — Data Sharing Statement [file jamanetwopen-e2251177-s002.pdf]

Data Sharing Statement

Au. Association of Sodium-Glucose Cotransporter 2 Inhibitor vs Dipeptidyl Peptidase-4 Inhibitor Use With Risk of Incident Obstructive Airway Disease and Exacerbation Events Among Patients With Type 2 Diabetes in Hong Kong. *JAMA Netw Open*. Published January 17, 2023. doi:10.1001/jamanetworkopen.2022.51177

Data
Data available: No

Additional Information
Explanation for why data not available: Restrictions apply to the availability of all data generated or analyzed during this study to preserve patient confidentiality. They were used under license by the Hospital Authority (HA) of Hong Kong and were not publicly available.
